# Protective effects of tanshinone IIA on endothelial progenitor cells injured by tumor necrosis factor-α

**DOI:** 10.3892/mmr.2015.3969

**Published:** 2015-06-22

**Authors:** XING-XIANG WANG, JIN-XIU YANG, YAN-YUN PAN, YE-FEI ZHANG

**Affiliations:** 1 Department of Cardiology, The First Affiliated Hospital, School of Medicine, Zhejiang University, Hangzhou, Zhejiang 310003, P.R. China; 2 Department of Cardiology, The First Affiliated Hospital, Zhejiang Chinese Medical University, Hangzhou, Zhejiang 310006, P.R. China; 3 Department of Emergency, The First Affiliated Hospital, School of Medicine, Zhejiang University, Hangzhou, Zhejiang 310003, P.R. China

**Keywords:** endothelial progenitor cell, migration, paracrine, tanshinone IIA, tumor necrosis factor-α, vasculogenesis

## Abstract

Tanshinone IIA (Tan IIA) is a Traditional Chinese Medicine commonly used in Asian and Western countries for the prevention and treatment of cardiovascular disorders, such as atherosclerosis. Endothelial dysfunction and associated inflammatory processes have a critical role in the development of atherosclerosis. Endothelial progenitor cells (EPCs) have been demonstrated to be involved in certain aspects of the endothelial repair process. The present study aimed to investigate the putative protective effects of Tan IIA on EPCs injured by tumor necrosis factor-α (TNF-α). The potential effects of Tan IIA on TNF-α-stimulated EPC proliferation, migration, adhesion, *in vitro* tube formation ability and paracrine activity were investigated in the current study. The results indicated that TNF-α impaired EPC proliferation, migration, adhesion capacity and vasculogenesis ability *in vitro* as well as promoted EPC secretion of inflammatory cytokines, including monocyte chemoattractant protein-1 (MCP-1), interleukin-6 (IL-6) and soluble CD40 ligand (sCD40L). However, Tan IIA was able to reverse these effects. In conclusion, these findings demonstrated that Tan IIA may have the potential to protect EPCs against damage induced by TNF-α. Therefore, these results may provide evidence for the pharmacological basis of Tan IIA and its potential use in the prevention and treatment of early atherosclerosis associated with EPC and endothelial damage.

## Introduction

Danshen is a type of Traditional Chinese Medicine, which has been commonly used in Asian and Western countries for the treatment of numerous diseases, including cerebrovascular and coronary artery diseases (1,2). Danshen contains aqueous and lipid soluble fractions. The two active hydrophilic components of Danshen are salvianolic acid B and danshensu, whereas tanshinone IIA (Tan IIA) and cryptotanshinone are the two lipophilic components (3). Among these components, Tan IIA serves as a marker component to exert therapeutic effects. Numerous studies in animal models and human patients have demonstrated that Tan IIA may be an effective antioxidant for protecting against atherosclerosis, the pathological basis for most clinical cardiovascular diseases (4,5). Endothelial dysfunction and associated inflammatory processes have been confirmed to have a critical role in the development of atherosclerosis (6). Therefore, maintaining the integrity of the vascular endothelium is essential for the prevention and treatment of early atherosclerosis.

Endothelial progenitor cells (EPCs) comprise a cell population that are able to circulate as well as proliferate and differentiate into mature endothelial cells (ECs). These cells do not express markers which are characteristic of mature endothelial cells and are not involved in lumen formation (7,8). Increasing evidence has suggested that EPCs have a role in the endothelial repair process through differentiation into mature ECs and the release of protective paracrine factors, including vascular endothelial growth factor (VEGF) and epidermal growth factor (EGF) (9). Therefore, improving EPC number and function through pharmacological modulation may be a novel strategy for atherosclerosis prevention and treatment.

Li *et al* reported that incubation of EPCs with salvianolic acids increased EPC number as well as enhanced the migratory, adhesive and vasculogenesis capacities of EPCs *in vitro* (10). Tan IIA is a primary active constituent of Danshen and is involved in mediating the beneficial actions of Danshen. However, there is little evidence for the effect of Tan IIA on EPC number and functions. In addition, tumor necrosis factor-α (TNF-α), a pro-inflammatory cytokine released in response to pathological conditions, is elevated in atherosclerosis and may promote its pathogenesis (11). TNF-α was demonstrated to reduce the proliferation, migration, adhesion and tube formation capacity of EPCs (12). However, the effect of Tan IIA on the functions of EPCs exposed to TNF-α remains to be elucidated. The present study aimed to examine the effects of Tan IIA on TNF-α-treated EPC number, adhesion, migration, tube formation capacity *in vitro* and paracrine function.

## Materials and methods

### Isolation and cultivation of EPCs

All experimental procedures involving animals were conducted in accordance with the Guide for the Care and Use of Laboratory Animals published by the National Institute of Health (Bethesda, MA, USA) and the present study was approved by the Institutional Animal Care Committee of Zhejiang University. A total of 40 male Sprague-Dawley rats (6–7 weeks old; 200 g) were provided by the Laboratory Animal Center of Zhejiang Province (Hangzhou, China), were housed in ten laboratory animal cages and maintained at a controlled temperature (20–22°C) and humidity (50–60%) with a 12 h light/dark cycle. The animals were provided with a standard diet and water *ad libitum*.

Expansion of rat bone marrow-derived EPCs was performed *in vitro*, as previously described (13,14). In brief, EPCs were obtained from the femurs of male Sprague-Dawley rats. Muscles and the connective tissue were detached, and the epiphyses were removed. Bone marrow was harvested by inserting an 18-gauge syringe needle into one end of the bone shaft and flushing the contents into a 60-mm culture dish consisting of endothelial basal medium-2 (EBM-2, Lonza, Basel, Switzerland) supplemented with 5% screened fetal bovine serum (FBS; Gibco Life Technologies, Carlsbad, CA, USA). Density gradient centrifugation for 20 min at 2,000 × g was then used to obtain mononuclear cells (MNCs) fraction. Cells were suspended in EBM-2 supplemented with 10% FBS and plated onto six-well plates (Corning, Inc., Tewksbury, MA, USA). Following 24 h, non-adherent cells were aspirated and transferred to separate plates for a further 24 h, following which the procedure was repeated again in order to rapidly remove adherent mature ECs and hematopoietic cells, which may be present in the aspirate. Non-adherent cells harvested at 48 h were used for subsequent experiments; these cells were cultured in EBM-2 supplemented with microvascular endothelial cell growth medium-2 (EGM-2 MV) single aliquots containing 10% FBS (50 ml), VEGF (50 ml), epidermal growth factor (EGF; 0.5 ml), fibroblast growth factor-2 (2 ml), insulin-like growth factor-1 (0.5 ml) and ascorbic acid (0.5 ml) (Lonza). Following 4 days in culture at 37°C in 5% humidified CO_2_, non-adherent cells were removed by washing in phosphate-buffered saline (PBS; Keyi, Hangzhou, China) and media was replaced every 3 days for 14 days.

### EPC fluorescent staining

Following 14 days in culture, attached MNCs were subjected to fluorescent chemical detection in order to identify EPCs. Direct fluorescent staining was employed in order to detect the dual binding of 1,1-dioctadecyl-3,3,3,3-tetramethylindocarbocyanine (DiI)-labeled acetylated low-density lipoprotein (acLDL; Molecular Probes, Life Technologies, Carlsbad, CA, USA) and fluorescein isothiocyanate (FITC)-conjugated Ulex europaeus agglutinin (UEA)-I (Sigma-Aldrich, St. Louis, MO, USA). Cells were incubated with 2.4 *μ*g/ml acLDL at 37°C with 5% humidified CO_2_ and then were fixed with 2% parafor-maldehyde (Bogoo, Shanghai, China) for 10 min. Following washing, EPCs were reacted with 10 *μ*g/ml UEA-I for 1 h to detect lectin binding. A fluorescence microscope (magnification, ×200; BX51; Olympus, Tokyo, Japan) was then used to visualize samples; double-positive cells were identified to be EPCs.

### Cell proliferation assay

A 3-(4,5-dimethylthiazol -2-yl)-2,5-di-phenyltetrazolium bromide (MTT) assay was used to determine the effect of Tan IIA and TNF-α on EPC proliferation. Following culture for 7 days, cells were digested with 0.25% trypsin (Keyi, Hangzhou, China) and then cultured in EBM-2 containing 10% FBS in 96-well culture plate (200 *μ*l/well). Following culture for a further 48 h at 37°C with 5% humidified CO_2_, the supernatant was discarded through aspiration and serum-free EBM-2 was added. Tan IIA (0, 1, 5, 10 and 20 *μ*M; Sigma-Aldrich) was then added (2×10^4^ cells, 200 *μ*l/well) and incubated for 18 h at 37°C with 5% humidified CO_2_, following which EPCs of each well were treated with 10 ng/ml TNF-α (Sigma-Aldrich) and cultured for 6 h at 37°C with 5% humidified CO_2_. EPCs without any treatment served as the control group. Wells were then supplemented with 20 *μ*l MTT (5 g/l; Sigma-Aldrich) and incubated for a further 4 h at 37°C with 5% humidified CO_2_. The supernatant was aspirated and the EPCs preparation was mixed for 10 min with 150 *μ*l dimethyl sulfoxide (DMSO; Sigma-Aldrich). Optical density (OD) values were then measured at 490 nm with a microplate reader (Victor3; PerkinElmer, Inc., Waltham, MA, USA).

### Migration assay

The migration rate of EPCs was determined using a Transwell^®^ chamber (Greiner; Monroe, NC, USA) with 8-*μ*m pore filters. Trypsin (0.25%) was used to detach isolated cells. Centrifugation for 10 min at 1,000 × g was performed in order to harvest cells, which were then resuspended in 500 *μ*l EBM-2 and counted. Subsequently, 2×10^4^ EPCs were placed in the upper Transwell^®^ chamber. Serum-free EBM-2 with VEGF was placed in the lower compartment of the chamber and the chambers were incubated for 24 h at 37°C in 5% humidified CO_2_. Following incubation, the lower side of the filter was washed with PBS and a cotton wool swab was used to remove the remaining cells on the upper face. Transwell^®^ filters were then fixed with 2% paraformaldehyde. EPCs were stained with 0.1% crystal violet solution (Bogoo) and cells migrating into the lower chamber were counted in five randomly selected microscopic fields (magnification, ×200; BX51 microscope).

### EPC adhesion assay

Following incubation with Tan IIA and TNF-α, EPCs were washed with PBS and 0.25% trypsin was used to gently detached cells. Centrifugation for 10 min at 1,000 × g was then performed and cells were resuspended in EBM-2, an equal number of cells were then replated onto fibronectin-coated culture dishes (Corning, Inc.) and incubated at 37°C in 5% humidified CO_2_ for 30 min. Once the non-adherant cells were washed away with PBS, the adherent cells were counted by independent blinded investigators in five randomly selected microscopic fields (magnification, ×400; BX51 microscope).

### In vitro tube formation assay

A Matrigel^®^ assay (EMD Millipore, Billerica, MA, USA) was used, according to the manufacturer's instructions, in order to assess endothelial tube formation *in vitro*. In brief, ECMatrix™ solution (frozen at −20°C; EMD Millipore) was thawed on ice overnight, mixed with 10X ECMatrix™ diluent (EMD Millipore) and placed into a 96-well plate at 37°C for 1 h to allow the matrix solution to solidify. Subsequently, 2×10^4^ EPCs were harvested and replated onto the solidified matrix solution with 150 *μ*l EBM-2. Cells were incubated for 18 h at 37°C and then fixed with 2% paraformaldehyde. The lengths of enclosed tubes which formed within the network were measured from five randomly selected microscopic fields (magnification, ×200; BX51 microscope). The experiment was repeated five times.

### ELISA analyses

Following 7 days of EPC culture, adherent cells were collected, re-inoculated onto six-well culture dishes and cultured for a further 48 h, the supernatant was then discarded through aspiration and supplement-free EBM-2 was added. Tan IIA (0, 1, 5, 10 and 20 *μ*M) was then added (200 *μ*l/well) and incubated for 18 h, following which EPCs were treated with 10 ng/ml TNF-α per well and cultured for 6 h. EPCs without treatment served as the control group. The original medium was discarded and cells were re-incubated with fresh EBM-2 with no supplement (0.5 ml/well). The protein levels of monocyte chemoat-tractant protein-1 (MCP-1), interleukin-6 (IL-6) and soluble CD40 ligand (sCD40L) were determined in cell culture supernatants using the MCP-1, IL-6 and sCD40L ELISA kits (R&D Systems, Minneapolis, MN, USA) according to the manufacturer's instructions.

### Statistical analysis

Values are presented as the mean ± standard deviation. Differences between group means were assessed by one-way analysis of variance for multiple comparisons followed by the Least Significant Difference post hoc test using SPSS 16.0 software (SPSS, Inc., Chicago, IL, USA). P<0.05 was considered to indicate a statistically significant difference between values.

## Results

### Characterization of EPCs

**Figure 1:**
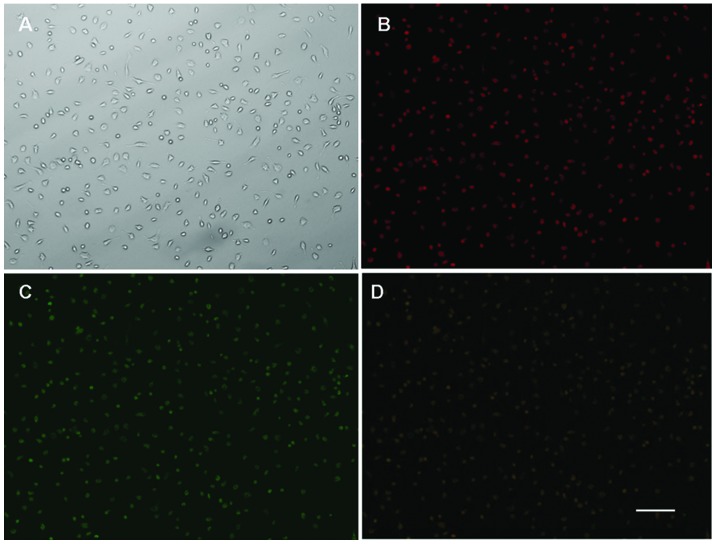
Immunofluorescence identification and immunophenotype of bone marrow derived-EPCs. (A) Attached cells exhibited a spindle shaped, endothelial cell-like morphology. (B) Adherent cells 1,1-dioctadecyl-3,3,3,3-tetramethylindocarbocyanine (DiI)-labeled acetylated low-density lipoprotein uptake (red; excitation wave-length, 543 nm) and (C) lectin binding (green; excitation wave-length, 477 nm) were assessed under a fluorescence microscopy. (D) Double positive cells (yellow; overlay of B and C) were identified as differentiating EPCs. Magnification, ×200; scale bar, 100 *μ*m. EPCs, endothelial progenitor cells.

MNCs isolated from rat bone marrow and cultured for 14 days, resulted in cells with a spindle-shaped, EC-like morphology (Fig. 1A). EPCs were confirmed to be adherent cells due to their double positive staining for DiLDL uptake and lectin binding, as determined using fluorescence microscopy (Fig. 1B–D).

### Effects of Tan IIA on proliferation of TNF-α-stimulated EPCs

**Figure 2:**
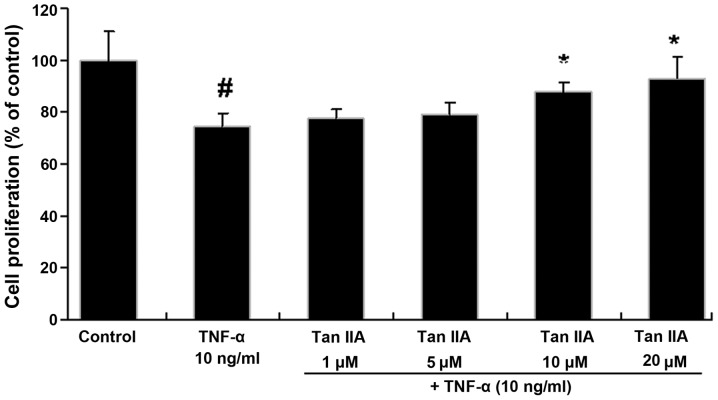
Proliferation of EPCs in response to TNF-α and Tan IIA, as determined using an MTT assay. EPC proliferation was decreased by TNF-α and restored by Tan IIA treatment. EPCs without treatment served as the control group. Values are presented as the mean ± standard deviation (n=5). ^#^P<0.01 vs. control group; ^*^P<0.05 vs. TNF-α group. EPCs, endothelial progenitor cells; TNF-α, tumor necrosis factor-α; Tan IIA, tanshinone IIA.

The effect of Tan IIA on EPC proliferation was assessed using the MTT assay (Fig. 2). TNF significantly reduced the proliferative activity of EPCs (74.59±5.05%) compared with that of the control (P<0.01). By contrast, this effect was reversed by Tan IIA in a dose-dependent manner, with significant increases at 10 and 20 *μ*M (87.88±3.58% and 93.09±8.12%, respectively) compared with the TNF-α-treated cells (P<0.05).

### Effects of Tan IIA on migration of TNF-α-stimulated EPCs

**Figure 3:**
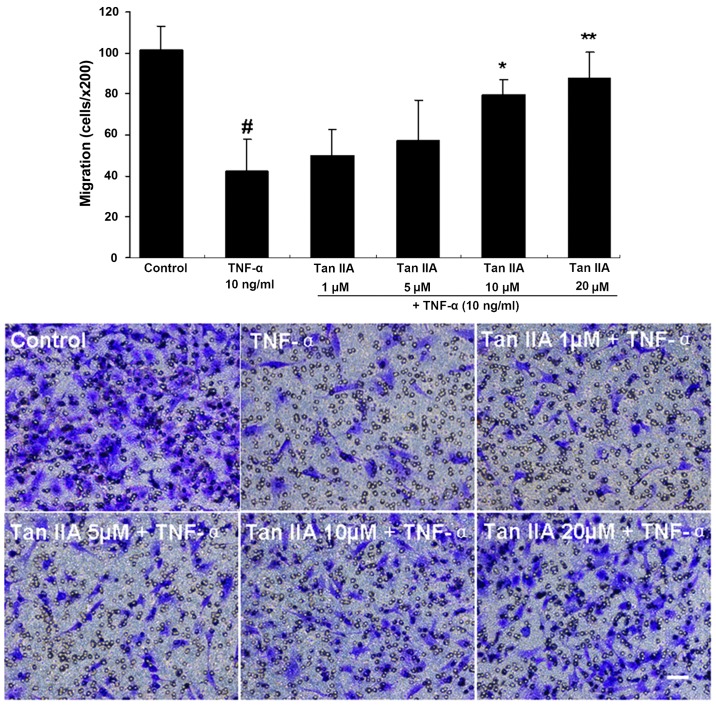
Migration of EPCs cocultured with TNF-α and Tan IIA, as determined by a Transwell^®^ assay. Migrating cells were counted in five randomly selected fields of vision. Migratory effect of EPCs was impaired by TNF-α, while this was reversed by Tan IIA treatment in a dose-dependent manner. Scale bar, 100 *μ*m. EPCs without treatment served as the control group. Data are presented as the mean ± standard deviation (n=5), magnification ×200. ^#^P<0.01 vs. control group; ^*^P<0.05 vs. TNF-α group; ^**^P<0.01 vs. TNF-α group. EPCs, endothelial progenitor cells; TNF-α, tumor necrosis factor-α; Tan IIA, tanshinone IIA.

A Transwell^®^ chamber assay was used to determine the effect of Tan IIA on EPC migration (Fig. 3). The results revealed that TNF-α decreased EPC migration compared with that of the control (42.4±15.3 vs. 101.2±12.2 cells/high-power field, respectively; P<0.01). In addition, Tan IIA dose-dependently increased EPC migration, which became significant at 10 *μ*M Tan IIA (79.6±7.3 cells/high-power field) compared with the TNF-α-treated cells (P<0.05), with the maximum migration rate following TNF-α treatment at 20 *μ*M Tan IIA (87.8±12.7 cells/high-power field; P<0.01).

### Effects of Tan IIA on the adhesion of TNF-α-stimulated EPCs

**Figure 4:**
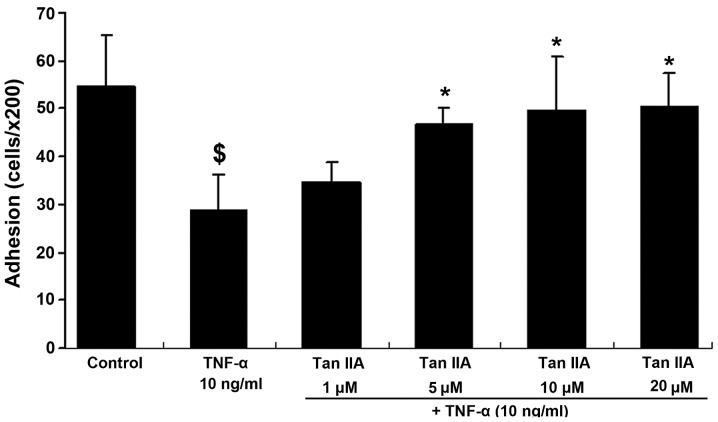
Adhesion capacity of EPCs treated with TNF-α and Tan IIA and plated onto fibronectin-coated culture dishes. TNF-α decreased EPC adhesion capacity, while Tan IIA dose-dependently increased cell adhesion. EPCs without treatment served as the control group. Data are presented as the mean ± standard deviation (n=5). ^$^P<0.05 vs. control group; ^*^P<0.05 vs. TNF-α group. EPCs, endothelial progenitor cells; TNF-α, tumor necrosis factor-α; Tan IIA, tanshinone IIA.

In order to determine whether Tan IIA altered the adhesion of cultured EPCs induced by TNF-α, EPCs were incubated with TNF-α (10 ng/ml) and Tan IIA (0, 1, 5, 10 and 20 *μ*M) for 24 h and then replated onto fibronectin-coated dishes. As shown in Fig. 4, EPCs pre-exposed to TNF-α exhibited a significant decrease in the number of adhesive cells compared with the control (28.8±7.6 vs. 54.6±10.8 cells/high-power field, respectively; P<0.05). By contrast, EPC adhesion ability was promoted by Tan IIA in a dose-dependent manner, with significant results observed at 5, 10 and 20 *μ*M (46.8±3.4, 49.6±11.3 and 50.4±7.0, respectively; P<0.05).

### Effects of Tan IIA on tube formation of TNF-α-stimulated EPCs in vitro

**Figure 5:**
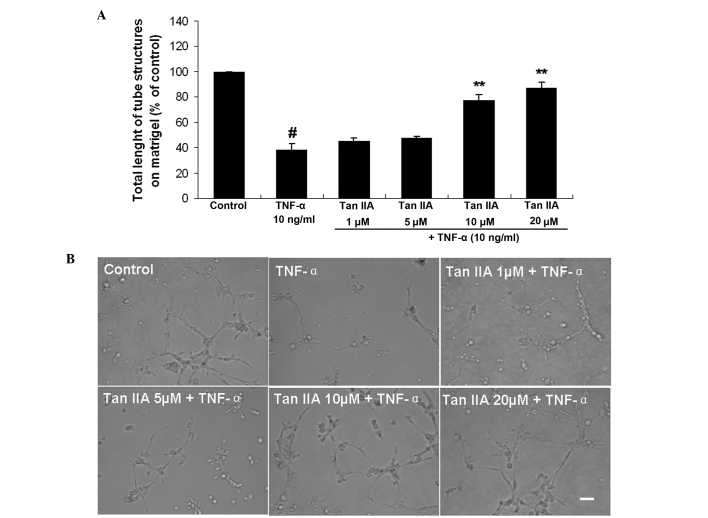
Effect of TNF-α and Tan IIA on EPC vasculogenesis ability *in vitro*. (A) Quantitative analysis of total length of the tubes formed on Matrigel^®^ for each experimental group. (B) Light micrographs showing typical tubules (magnification, ×200; scale bar, 100 *μ*m). TNF-α impaired EPC vasculogenesis ability *in vitro*, while Tan IIA dose-dependently increased cell vasculogenesis *in vitro*. EPCs without treatment served as the control group. Data are presented as the mean ± standard deviation (n=5). ^#^P<0.01 vs. control group; ^**^P<0.01 vs. TNF-α group. EPC, endothelial progenitor cells; TNF-α, tumor necrosis factor-α; Tan IIA, tanshinone IIA.

EPCs were incubated overnight in starvation medium and then stimulated for 24 h with TNF-α and Tan IIA in basal medium. The lengths of enclosed tubes which formed within the network were measured. As shown in Fig. 5, TNF-α significantly impaired EPC vasculogenesis *in vitro* (38.50±4.48%) compared with the control (P<0.01). Tan IIA restored EPC vasculogenesis ability *in vitro* in a dose-dependent manner following TNF-α treatment, with significant results at 10 and 20 *μ*M Tan IIA (77.86±3.93 and 87.39±3.95%, respectively) compared with the TNF-α-treated cells (P<0.01).

### Effects of Tan IIA on paracrine function of TNF-α-stimulated EPCs

**Figure 6:**
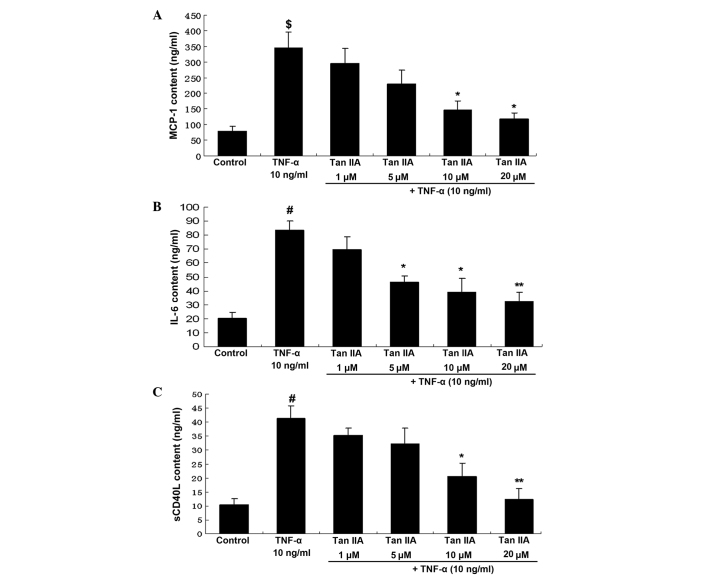
Effects of TNF-α and Tan IIA on EPC paracrine function. ELISAs were used to determine the effect of TNF-α and Tan IIA on (A) MCP-1, (B) IL-6 and (C) sCD40L content of EPCs. TNF-α increased EPC secretion of MCP-1, IL-6 and sCD40L, while Tan IIA dose-dependently decreased cell paracrine function. EPCs without treatment served as the control group. Data are presented as the mean ± standard deviation (n=3). ^$^P<0.05 vs. control group; ^#^P<0.01 vs. control group; ^*^P<0.05 vs. TNF-α group; ^**^P<0.01 vs. TNF-α group. EPC, endothelial progenitor cells; TNF-α, tumor necrosis factor-α; Tan IIA, tanshinone IIA; MCP-1, monocyte chemoattractant protein-1 (MCP-1); IL-6, interleukin-6; sCD40L, soluble CD40 ligand.

The effect of Tan IIA on EPC paracrine activity was assessed using ELISA analyses (Fig. 6). Compared with the control group, TNF-α significantly enhanced the protein levels of MCP-1 in the EPC supernatant compared with the control group (345.2±51.0 vs. 79.8±15.2 ng/ml, respectively; P<0.05). By contrast, Tan IIA dose-dependently decreased EPC secretion of MCP-1, with significant results at 10 and 20 *μ*M Tan IIA (146.5±28.4 and 116.9±21.1 ng/ml, respectively) compared with the TNF-α-treated cells (P<0.05). In addition, TNF-α significantly increased IL-6 levels in the EPC supernatant compared with the control group (83.5±6.5 vs. 20.4±4.2 ng/ml; P<0.01). Tan IIA dose-dependently decreased EPC secretion of IL-6, with significant results at 5 *μ*M (46.4±4.2 ng/ml; P<0.05) and a maximum inhibition observed at 20 *μ*M (32.3±6.6 ng/ml; P<0.01) compared with the TNF-α-treated cells. Furthermore, TNF-α significantly augmented sCD40L levels in the EPC supernatant compared with the control group (41.3±4.5 vs. 10.4±2.2 ng/ml; P<0.05). By contrast, Tan IIA dose-dependently decreased EPC secretion of sCD40L, with significant results at 10 *μ*M (20.6±4.8 ng/ml; P<0.05) and 20 *μ*M (12.4±3.9 ng/ml; P<0.01) compared with the TNF-α-treated cells.

## Discussion

The vascular endothelium is located at the interface between blood and tissue and has a crucial role in the maintenance of vessel wall integrity (15). Numerous environmental factors have been reported to damage the structure and function of the endothelium, resulting in the development of various diseases (15). It has been confirmed that endothelial dysfunction has a critical pathogenic role in various diseases, including atherosclerosis, hypertension, diabetes and thrombosis (16). Endothelial dysfunction may induce subsequent pathogenic events, such as inflammation and thrombosis. It was reported that inflammation was involved at all stages of atherosclerosis, including lesion formation and plaque stability (17). Thus, it was suggested that endothelial dysfunction may be a major promoter of atherosclerosis (18). In order to prevent the pathogenesis and development of atherosclerosis, it is essential to repair endothelial dysfunction. Endothelial dysfunction occurs due to an imbalance between the severity of injury and the ability of the endothelium to repair (19). Increasing evidence has suggested that EPCs are involved in certain aspects of this repair process (20–22). In an animal study, EPCs were demonstrated to contribute to vessel formation through differentiation into mature ECs and incorporation into the vessel wall (20). Other studies have reported that accelerated re-endothelialization by EPCs effectively inhibited smooth muscle cell proliferation, migration and neointima formation, therefore preventing the initiation and development of the early stages of restenosis following vascular injury (21,22). EPCs have an essential role in the prevention of early atherosclerosis and the treatment of restenosis following angioplasty (23).

Tan IIA, a major lipid-soluble active compound of Danshen, was demonstrated to have anti-atherosclerotic and anti-inflammatory properties (24). Therefore, Tan IIA may be a promising candidate for the development of novel therapeutic strategies for the prevention and treatment of atherosclerosis (25). *In vitro* studies have indicated that Tan IIA may down-regulate the expression of intercellular adhesion molecule-1 in TNF-α-induced human umbilical vein endothelial cells as well as inhibit the oxidation of low-density lipoprotein (26,27). Considering the close associations between EPCs and ECs, it was hypothesized that Tan IIA may exert anti-atherosclerotic properties through the protection of EPC function. The present study aimed to examine the effect of Tan IIA on TNF-α-treated EPC number, adhesion, migration, tube formation capacity *in vitro* and paracrine activity. To the best of our knowledge, the current study was the first report of the effects of Tan IIA on the function of EPCs stimulated by TNF-α.

The present study demonstrated that EPC proliferation, migration, adhesion capacity and vasculogenesis ability *in vitro* were impaired by 10 ng/ml TNF-α. TNF-α is a pro-inflammatory cytokine that is released in atherosclerosis and has been reported to promote the process of atherosclerosis (11). The results of the present study suggested that TNF-α may participate in the process of atherosclerosis by affecting EPC function; therefore, inhibiting these functions may be a promising therapeutic strategy for the treatment of atherosclerosis. The current study demonstrated that Tan IIA was able to significantly improve EPC function, as demonstrated by its capacity to increase EPC proliferation, migration and adhesion. These observations led to the subsequent investigation of the role of Tan IIA in promoting angiogenesis. A vasculo-genesis assay *in vitro* demonstrated that Tan IIA upregulated vasculogenesis in a dose-dependent manner, with significant results at concentrations of 10 and 20 *μ*M Tan IIA.

Increasing evidence has supported the involvement of inflammation in the pathogenesis and development of atherosclerosis (28,29). It was reported that the inflammatory responses mediated by cytokines, including MCP-1, IL-6 and sCD40L, were important in atherosclerosis (30). In the current study, the effects of Tan IIA on EPC secretion of MCP-1, IL-6 and sCD40L following stimulation by TNF-α, were investigated. The results demonstrated that TNF-α enhanced the release of MCP-1, IL-6 and sCD40L by EPCs. However, treatment with Tan IIA reduced the levels of IL-6, sCD40L and MCP-1 in the EPC supernatant.

MCP-1 is a pro-inflammatory chemokine, which has been reported to promote vascular inflammation and atherosclerosis (31,32). IL-6 is an inflammatory cytokine of circulation, the levels of which were shown to reactively rise with coronary heart disease; in addition, the IL-6 pathway was suggested to have a role in the process of atherosclerosis (33). CD40 and CD40 ligand activation are known to be important inflammatory signals in atherosclerosis (17). Previous *in vivo* and *in vitro* studies have demonstrated that Tan IIA exerts protective effects in atherosclerosis through decreasing the expression of CD40 (34,35). In the present study, the release of the pro-atherogenic chemokines MCP-1, IL-6 and sCD40L was markedly reduced by coculturing the cells with Tan IIA. Therefore, the present study demonstrated that compared with the control group, Tan IIA significantly improved cell function as well as reduced the protein levels of MCP-1, IL-6 and sCD40L in the EPC supernatant. These results suggested that Tan IIA may not only be used to reduce the damage of EPCs from TNF-α, but also to significantly enhance the anti-inflammatory ability of EPCs.

Of note, in the present study EPCs were cultured with MNCs derived from healthy rats in order to investigate the protective effects of Tan IIA on EPCs in the presence of TNF-α. The findings suggested that Tan IIA may exert therapeutic effects on endothelial dysfunction-associated diseases, such as atherosclerosis, through the protection of EPC function. However, in order to further study the anti-atherosclerotic effects of Tan IIA, future studies should use MNCs derived from atherosclerotic animals.

In conclusion, the results of the present study demonstrated that TNF-α impaired EPC proliferation, migration, adhesion capacity and vasculogenesis ability *in vitro*, as well as promoted EPC secretion of inflammatory cytokines, including MCP-1, IL-6 and sCD40L. In addition, it was revealed that Tan IIA was able to reverse these effects. Therefore, treatment with Tan IIA may have the potential to protect EPCs against damage induced by TNF-α. These results may provide evidence for the pharmacological basis of Traditional Chinese Medicine in the prevention and treatment of early atherosclerosis associated with EPC and endothelial damage.
